# Access and use of sexual and reproductive health services among asylum-seeking and refugee women in high-income countries: A scoping review

**DOI:** 10.1371/journal.pone.0312746

**Published:** 2024-11-07

**Authors:** Emma Stirling-Cameron, Salma Almukhaini, Justine Dol, Benjamin J. DuPlessis, Kathryn Stone, Megan Aston, Shira M. Goldenberg

**Affiliations:** 1 School of Population and Public Health, University of British Columbia, Vancouver, Canada; 2 Aligning Health Needs & Evidence for Transformative Change: An Affiliated Centre of the Joanna Briggs Institute, Dalhousie University, Halifax, Canada; 3 School of Nursing, Dalhousie University, Halifax, Canada; 4 College of Nursing, Sultan Qaboos University, Muscat, Oman; 5 Centre for Pediatric Pain Research, IWK Health, Halifax, Canada; 6 Faculty of Medicine, University of British Columbia, Vancouver, Canada; 7 Department of Social Dimensions of Health, University of Victoria, Victoria, Canada; 8 School of Public Health, San Diego State University, San Diego, CA, United States of America; Universiti Pertahanan Nasional Malaysia, MALAYSIA

## Abstract

**Background:**

Refugee and asylum-seeking women are known to experience a myriad of intersecting sociocultural, institutional, and systemic barriers when accessing healthcare services after resettlement in high-income countries. Barriers can negatively affect service uptake and engagement, contributing to health inequities and forgone care. Access to sexual and reproductive healthcare (e.g., family planning, cervical cancer prevention) has largely been understudied. This scoping review sought to: i) examine the use of sexual and reproductive health services among refugee and asylum-seeking women in high-income countries; and ii) identify barriers and facilitators influencing access to sexual and reproductive healthcare for refugee and asylum-seeking women in high-income countries.

**Methods:**

This review was conducted in accordance with Joanna Briggs Institute Methodology for Scoping Reviews. Ten databases (e.g., CINAHL, MEDLINE, Embase) were searched for qualitative, quantitative, mixed method studies, and gray literature published anytime before February 2024 across high-income countries (defined by the World Bank). The Health Behaviour Model was used to examine and understand factors influencing service use and access.

**Results:**

3,997 titles and abstracts were screened, with 66 empirical studies included. Most were conducted in the United States (44%), Australia (25%), Europe (18%) and elsewhere and were qualitative (68%). Papers largely addressed contraception, abortion, cervical cancer screening, gender-based violence, and sexual health education. Included studies indicated that refugee and asylum-seeking women in high-income countries face a greater unmet need for contraception, higher use of abortion care, and lower engagement with cervical cancer screening, all when compared to women born in the resettlement country. Frequently reported barriers included differences in health literacy, shame and stigma around sexual health, language and communication challenges, racial or xenophobic interactions with healthcare providers, and healthcare/medication costs.

**Conclusions:**

Studies across the globe identified consistent empirical evidence demonstrating health inequities facing refugee and asylum-seeking and myriad intersecting barriers contributing to underuse of essential sexual and reproductive health services. Facilitators included multilingual healthcare provider, use of interpreters and interpretation services, community health promotion work shops, and financial aid/Medicare.

## Introduction

Sexual and reproductive health (SRH) is a fundamental component of all persons’ well-being and quality of life [1]. Sexual and reproductive health is defined as “a state of physical, emotional, mental, and social wellbeing in relation to all aspects of sexuality and reproduction, not merely the absence of disease, dysfunction, or infirmity.” [1] ^(p.2646)^ Access to SRH reduces the incidence of gender-based violence, and helps to prevent unplanned pregnancy, reproductive cancers, unsafe abortion, and sexually transmitted and blood borne illnesses (STBBIs), ultimately contributing to gender equality, social justice, and economic development [1]. The 2018 Lancet-Guttmacher Institute report on SRH identified refugee populations as at-risk for worsened SRH outcomes, and suggested that this population is in need of special attention [1]. For the purposes of this review, SRH does not include perinatal healthcare, as this has been reviewed extensively elsewhere [2].

The world is currently amid the largest forced migration crisis ever recorded [3]. Increasing effects of the climate crisis, civil conflict, war, chronic poverty, and political instability are forcibly displacing thousands of people per day. The United Nations High Commissioner for Refugees estimated that 117.3 million people were currently displaced across the globe at the end of 2023, including 43.4 million refugees and 6.9 million asylum-seekers [4]. Approximately 1.7% of the world’s refugee population is housed in high-income countries, including the United States, Canada, Australia, and Germany [4]. Two-thirds of those forcibly displaced are fleeing five countries: Syria, Venezuela, Afghanistan, South Sudan, and Myanmar; the vast majority are racialized, and have been exposed to violence, conflict, and other traumas [4].

Half of all refugees and asylum-seekers are women (4). In situations of armed conflict, instability, and forced displacement, the breakdown of social infrastructures, disintegration of families and community, and high rates of poverty create a context in which women are particularly vulnerable [5, 6]. Women are exposed to extremely high rates of gender-based violence and human rights violations, including sexual abuse and exploitation, human trafficking, intimate partner violence, and child marriage [7, 8]. Single mothers, unaccompanied minors, and transgender women often face abuse and exploitation at the hands of intimate partners, people smugglers, humanitarian workers, and law enforcement agents [5–7, 9]. High rates of violence, coupled with limited access to SRH in refugee camps and temporary settlements, has contributed to disparities in SRH among refugee and asylum-seeking women globally. This includes the transmission of STBBIs, including HIV/AIDS, risks of unwanted pregnancies, unsafe abortions, and the forgone detection and treatment of reproductive cancers [10]. Refugee and asylum-seeking women may be in particular need of accessible, affordable, safe, and trauma-informed SRH services upon arrival in resettlement/host countries [1].

While high-income nations boast quality, comprehensive healthcare systems, SRH disparities persist among refugee and asylum-seeking populations globally [11–14]. Individual or interpersonal socio-cultural and religious beliefs endorsing traditional and patriarchal gender roles and stigma around SRH may limit women’s autonomy and decision to engage in SRH services. Often defaulted as caregivers in times of crisis [5] women report giving priority to fulfilling basic needs, such as finding affordable housing and employment—putting the needs of their families above their own [15–17]. Direct access to healthcare services is limited by women’s ability to obtain childcare and find transportation to appointments, in addition to navigating the healthcare system in their host country [17, 18].

Health and social systems in majority-White countries are often plagued by systemic racism, fostering inequities and mistrust among racialized people, including many refugees and asylum-seekers [19, 20]. Moreover, language and communication differences and a lack of interpretation services or culturally and linguistically diverse staff can complicate service use and limit the uptake of treatment and educational resources [19, 20]. Lack of, or poor-quality insurance restricts access to all healthcare, but particularly to non-acute, preventative care services, such as cervical cancer screening and prevention, and STBBI screening. The high cost of prescriptions, including hormonal and long-acting contraceptives, has been reported as a deterrent to use [21].

The reduced accessibility of SRH services and supports has resulted in inequitable negative SRH outcomes among resettled refugee women in high-income countries, including unwanted pregnancy and abortion [11, 12, 14], lower than recommended rates of cervical cancer screenings [22] and HPV vaccinations [12], high rates of STBBIs [11], reduced uptake of contraception [23], and non-consensual or painful sex [12, 24, 25]. Previous systematic reviews have largely focused on perinatal and infant healthcare access and service use for refugee and asylum-seeking women in high-income countries [2, 26, 27], neglecting other major components of sexual and reproductive health (e.g., family planning, cervical cancer screening, abortion care). Others have focused on refugee camp settings; spaces which face unique challenges and resource constrains, which differ from health access in high-income countries [10, 28]. As refugee resettlement in high-income countries is only expected to rise, a comprehensive, global review of the existing literature is warranted to understand *access to and use of SRH services among refugee and asylum-seeking women across these nations*.

### Objectives

The objectives of this scoping review were to: i) examine the use of sexual and reproductive health services among refugee and asylum-seeking women in high-income countries; and ii) identify barriers and facilitators influencing access to sexual and reproductive healthcare for refugee and asylum-seeking women in high-income countries.

## Methods

The scoping review was conducted in accordance with Joanna Briggs Institute methodology for scoping reviews [29]. A scoping review protocol for this paper was published elsewhere [30]. A preliminary search of PROSPERO, MEDLINE, the Cochrane Database of Systematic Reviews, Open Science Frameworks, and the *JBI Database of Systematic Reviews and Implementation Reports* was conducted and no current or in-progress scoping reviews or systematic reviews on the topic were identified.

### Conceptual framework

We utilized Kaufmann et al. (2014)’s health behavior model [31] for sexual and reproductive healthcare, which has been adapted from Bronfenbrenner’s socio-ecological model [32]. The purpose of employing this conceptual framework was to organize and categorize review findings related to objective 2, which sought to identify barriers and facilitators influencing access to sexual and reproductive healthcare for refugee and asylum-seeking women in high-income countries. The health behaviour model has four nested categories which are used to describe factors influencing health care service access and use. These include individual (e.g., knowledge and information, stigma/shame), community (e.g., socio-cultural and religious norms, peer pressure, relationship equity, social support), institutional and health system (e.g., provider biases, health system operations), and structural level factors (e.g., poverty, transportation and infrastructure, service cost). Similar frameworks have been used in other scoping reviews related to refugee health [10, 33].

### Eligibility criteria

#### Participants

This review considered studies that focused on refugee or asylum-seeking women (also sometimes referred to as refugee-claimant or undocumented women) who have been resettled in or have fled to high-income countries. A refugee is defined as someone who has been forced to flee their country of origin due to conflict, persecution, or violence, and who cannot return to their home country [34]. An asylum-seeker is someone who has fled their home country in search of protection but may not fulfil the strict criteria outlined in Convention Relating to the Status of Refugees [35]. They have applied for refugee status and are awaiting a decision [36]. Immigrant and migrant women and undocumented persons were not included. Though there are potential similarities across groups, economic im/migrants often have additional legal protections not afforded to asylum seekers or refugees (depending on the country); whereas undocumented people often have even fewer protections and privileges, owing to their lack of status. As such, we believed there would be differences among these groups, and restricted our search to just asylum-seekers and refugees. However, many findings may apply to these groups as well. No restrictions were placed on country of origin, women’s age, length of time in their host country, religion, sexual orientation, whether they had children, or their marital status. Studies that elicited health care providers perceptions on access to SRH services for refugee women were also included. This included nurses, family physicians, obstetrician gynecologists, midwives, doulas, social workers, refugee resettlement workers, and other relevant key informants.

#### Concept

The main concept under study is access and use of SRH services for women. SRH services have been previously defined to include the provision of accurate information and counseling on SRH, including comprehensive, evidence-based sexuality education; information, counseling, and care related to sexual function and satisfaction; prevention, detection, and management of sexual- and gender-based violence and coercion; a choice of safe and effective contraceptive methods; safe and effective abortion services and care; prevention, management, and treatment of infertility; prevention, detection, and treatment of sexually transmitted infections, including HIV, and of reproductive tract infections; and prevention, detection, and treatment of reproductive cancers [1]. Access to health care was defined as “the opportunity to reach and obtain appropriate healthcare services in situations of perceived need for care.” (33, p.4). Access to health care has five key features, including the ability of the user to i) identify their health care needs, ii) seek out health care services, iii) reach necessary health care resources, iv) obtain or use health care services, and v) be offered services appropriate to the needs of care [37].

### Context

This review considered studies that describe the access and use of SRH of refugee and refugee-claimant women that have been resettled or are seeking asylum in high-income countries, defined based on the 2020 World Bank income classifications [38]. Studies which collected data in any of the listed countries were included in the review. Access to services in refugee camps, asylum-processing or detention centers, or other temporary settlements were not included as they are liable to differ from community resettlement settings.

### Types of sources

This scoping review considered all study designs: quantitative, qualitative, and mixed-methods. Systematic reviews were screened for potentially relevant papers. Any papers included in the review that appeared to fit study inclusion criteria were added to the study, but systematic review papers themselves were excluded. Due to resource constraints, studies were only included if they were available in English, French, or Arabic. There were no date restrictions.

### Search strategy

A JBI three-step search strategy was implemented in this review [29]. The search strategy, developed in cooperation with a librarian scientist, aimed to locate published and unpublished studies (see S1 Appendix for CINAHL search details). The text words contained in the titles and abstracts of relevant articles, and the index terms used to describe the articles were used to develop a full search strategy for CINAHL. The search strategy, including all identified keywords and index terms, was adapted for each included information source. The reference lists of articles selected for full-text review were screened for additional studies.

#### Information sources

Databases searched included CINAHL (EBSCO), MEDLINE (Ovid), Embase (Elsevier), Studies on Women and Gender Abstracts (Taylor and Francis), Academic Search Premier (EBSCO), Sociological Abstracts (ProQuest), Social Services Abstracts (ProQuest), PAIS Index (ProQuest), Public Affairs Index (EBSCO), and PsycINFO (American Psychological Association). The original search was conducted in February 2020 from database inception and updated in February of 2024. Sources of unpublished studies and gray literature included, The United Nations, the United Nations High Commissioner on Refugees, the International Organization for Migration, Centers for Disease Control, United Nations Population Fund, the World Health Organization, Google Scholar, ProQuest Dissertations and Theses Databases, Migration Policy Institute, Refugee Council, Canadian Council for Refugees, Gray Literature Report (via New York Academy of Medicine website), and Grey Source—a Selection of Web-based Resources in Grey Literature.

#### Study selection

Following the search, all identified records were collated and uploaded into Covidence (Veritas Health Innovation, Melbourne, Australia) and duplicates were removed through the built-in automation process. Titles and abstracts were screened by two independent reviewers for assessment against the inclusion criteria for the review. A document was developed by the primary author (ESC) detailing the PCC framework to be used to screen relevant articles. This was adapted based on feedback from all members of the review team during early phases of full-text screening and uploaded to the inclusion/exclusions section of Covidence. The full text of selected citations was assessed in detail against the inclusion criteria by two independent reviewers. Any disagreements that arose between the reviewers at each stage of the selection process were resolved through discussion or with a third reviewer. The results of the search have been presented in a Preferred Reporting Items for Systematic Reviews and Meta-analyses Extension for Scoping Reviews (PRISMA-ScR) flow diagram [39].

#### Data extraction and charting

We conducted a data charting process using the online extraction tools available through Covidence (Veritas Health Innovation, Melbourne, Australia) to sort the data and organize our findings. Our extraction tool collected evidence on study participants country of origin, ethnicity, migration status (e.g., refugee, asylum-seeker), country of resettlement, study method, main area of SRH under consideration, use of any SRH services, barriers to accessing care, facilitators to accessing care. This extraction tool was piloted by the study team, after which it was refined and finalized. Data from each full text article were extracted independently by two different reviewers (inclusive of authors ESC, SA, JD, BD, and MA). Data were then divided by objective and are reported below in two sections, each addressing an objective. Due to the large number of studies retrieved, the Health Behaviour Model was implemented to guide data analysis and reporting, which was not included in the original, published study protocol.

## Results

### Study inclusion

**Fig 1:**
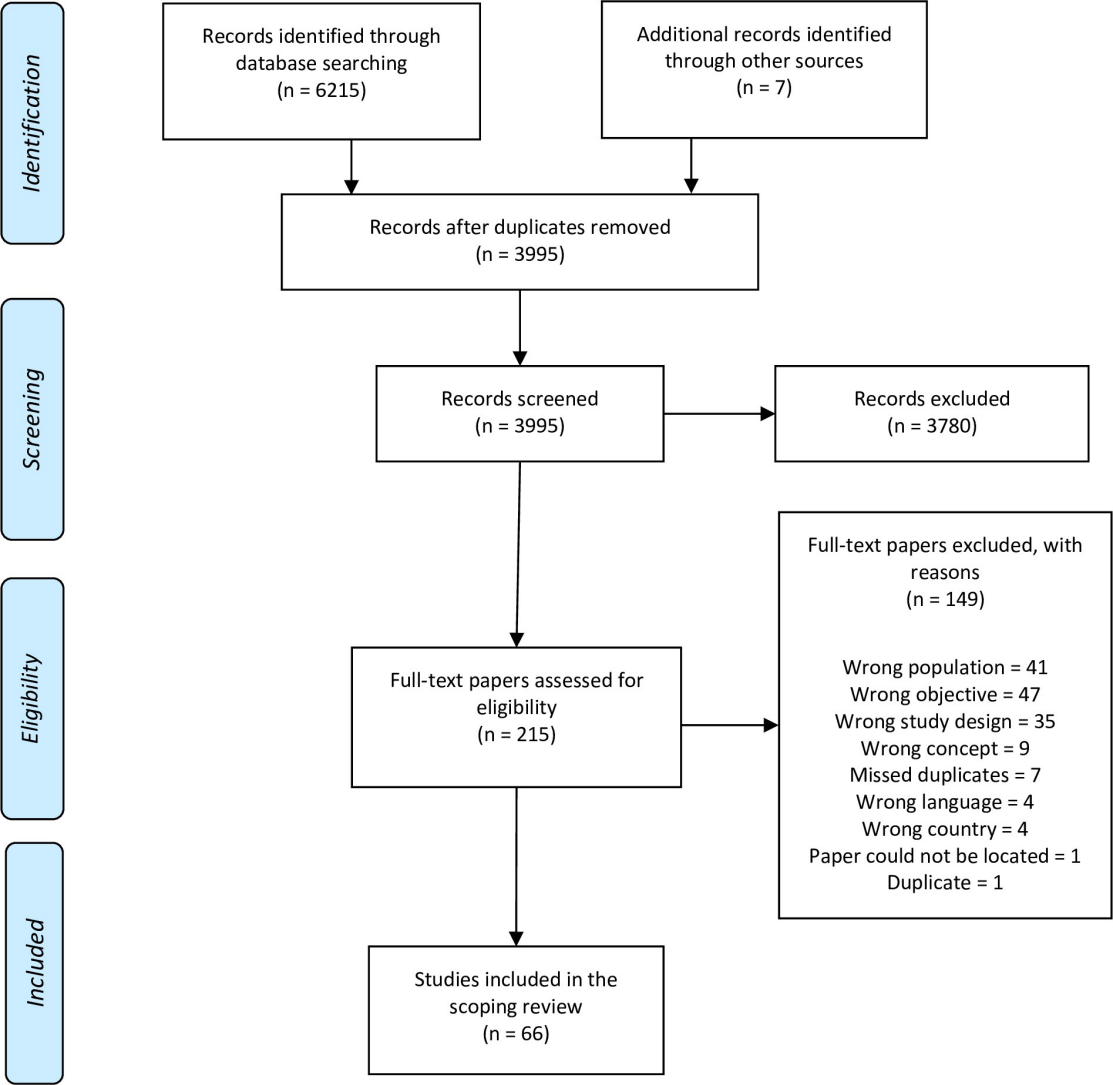
PRISMA flowchart: Search results, study selection, and inclusion process [39].

Details of included and excluded studies are reported in Fig 1. The initial search of 10 databases and hand-searching revealed a total of 6222 articles. 2227 duplicates were removed, and 3995 articles underwent title and abstract screening. 215 articles progressed to full-text review, of which 66 articles fit study inclusion criteria and were included in data extraction.

### Characteristics of included studies

A total of 66 studies were included in this review. Full study characteristics can be seen in Table 1. Most studies were qualitative (n = 45), followed by quantitative (n = 17) and mixed method designs (n = 4). Most studies were published in the United States (n = 29) [40–68] followed by Australia (n = 17) [13, 69–84] Canada (n = 4) [85–88] Switzerland (n = 3) [89–91], Canada and Australia together (n = 2) [12, 92] Sweden (n = 2) [93, 94] the Netherlands (n = 2) [95, 96] United Kingdom (n = 2) [97, 98] Finland (n = 1) [99] Israel (n = 1) [100] Norway (n = 1) [101], Germany (n = 1) [23], and South Korea (n = 1) [102]. Most studies focused on understanding the experiences of resettled refugee women (n = 41), followed by asylum-seeking women (n = 6), healthcare or service providers (n = 12), refugees and healthcare providers (n = 5), and refugees and asylum-seekers (n = 2).

**Table 1:** Characteristics of included studies on access to or use of sexual and reproductive healthcare services among refugee or asylum-seeking women in high-income countries (n = 61).

First listed author (year)	Location	Methodology	Participant ethnicity or country of origin	Participant population	SRH area under study	Objective
Agbemenu (2020)	USA	Quantitative, cross-sectional, convenience survey	DR Congo (50%), Somalia (30%), Kenya (9%), other African countries	Resettled refugees	Contraception, abortion, & family planning	What are African refugee women’s family planning attitudes (pregnancy intention, desired timing, and perceived fecundity)? What are African refugee women’s family planning behaviors (use vs. non-use of methods, type of methods used)?
Allen (2019)	USA	Qualitative, thematic analysis, focus groups	Somali	Resettled refugees	Cervical cancer screening & prevention	To explore facilitators and barriers to cervical cancer screening and human papilloma virus (HPV) vaccination among Somali refugee women and their children
Aptekman (2014)	Canada	Quantitative, retrospective chart review	63.5% European, 13.5% Africa, 11.5% Asia, 11.5 South/Latin America	Resettled refugees	Contraception, abortion, & family planning	To describe what women of reproductive age who received primary care at a refugee health clinic were using for contraception upon arrival to the clinic, and to quantify the unmet contraceptive needs within that population
Anstiss (2023)	Australia	Qualitative, thematic analysis using help-seeking framework	Afghanistan, Syria, and Africa (Liberia, Burundi, Kenya, Sierra Leone, Somalia and Sudan)	Resettled refugees	Gender-based violence	To: (a) determine the nature and scope of help-seeking among participants; (b) investigate the individual, interpersonal and sociocultural influences on help-seeking; and; (c) provide suggestions to inform preven-tion and intervention efforts
Babatunde-Sowole (2020)	Australia	Qualitative, oral narratives derived from two primary qualitative datasets underwent secondary thematic analysis	West African	Resettled refugees	Cervical cancer screening & prevention	To explore the beliefs, understandings, and use of health and healthcare screening services among African refugee women
Barnes (2004)	USA	Quantitative, retrospective chart review	31% Cuban, 26% Bosnian, 25% Vietnamese, and 18% other ethnicities.	Resettled refugees	Cervical cancer screening & prevention	To describe reproductive health needs and screening rates for breast and cervical cancer for newly arrived (less than 90 days) refugee women
Bartlett (2020)	Australia	Qualitative, group surveys, photovoice, GIS go-along	Iraq (78.57%), Syria (10.71%), Egypt (3.57%) and Ethiopia (3.57%)	Resettled refugees	Cervical cancer screening & prevention	This community-based participatory research focused on physical and social barriers to healthcare for refugee women
Byrskog (2015)	Sweden	Qualitative, interviews, thematic analysis	Somali	Healthcare providers	Gender-based violence	The aim was to explore ways ANC midwives in Sweden work with Somali born women and the questions of exposure to violence
Casillas (2015)	Switzerland	Quantitative, secondary analysis using cross-sectional data	Latin America, Asia, Europe, Africa	Refugee-claimants	Contraception, abortion, & family planning	To examine the effect of status on unintended pregnancy among women presenting at a Swiss public hospital in Geneva
Connor (2016)	USA	Quantitative, descriptive for quantitative and thematic coding for qualitative interviews	Somali	Resettled refugees	Gender-based violence	To explore sexual values, attitudes, and behaviors among Somali refugees
Critelli (2019)	USA	Qualitative; Interpretive and trauma informed methodologies; Thematic analysis	China, Eritrea, Turks and Caicos, Puerto Rico, Dominican Republic, Burma, Jamaica Argentina	Resettled refugees	Gender-based violence	To examine the service needs of women with immigrant and refugee status experiencing domestic violence
Dean (2017)	Australia	Quantitative; Parallel-mixed method study	Sudanese	Resettled refugees	SRH education; Contraception, abortion, & family planning	To explore the complex array of intergenerational social, cultural, and environmental variables influencing the sexual health knowledge, attitudes and behaviour of young Sudanese Queenslanders
Degni (2006)	Finland	Mixed-Methods; Survey; Interviews using content analysis	Somali	Resettled refugees	Contraception, abortion, & family planning	To assess attitudes towards and perceptions about contraceptive use among married refugee women of Somali descent
Dhar (2017)	USA	Qualitative; Semi-structured interviews (no methodology listed)	Bhutanese	Resettled refugees	Sexual health education	To conduct an exploratory study examining SRH attitudes/beliefs among un-married, female, Bhutanese, resettled refugee youth
Fineran (2020)	USA	Qualitative, Participatory Action Research	Iraq, Jordan, Morocco, Somalia, Sudan	Resettled refugees	Gender-based violence	To (a) document the narratives of Muslim refugee women on IPV, and (b) identify the barriers that stop refugee women from accessing culturally appropriate services
Gebreyesus (2020)	Israel	Qualitative; Some elements of grounded theory	Eritrean	Refugee-claimants	Contraception, abortion, & family planning	To explore the structural barriers to family planning (i.e. contraceptive services) that are rooted in their temporary legal status and the patchwork of family planning services
Ghebre (2015)	USA	Qualitative; Individual interviews (no methodology listed)	Somali	Healthcare providers speaking about former refugees	Cervical cancer screening & prevention	To examine barriers to and facilitators of cervical cancer screening among Somali immigrant women in Minnesota
Goosen (2009)	The Netherlands	Quantitative; Retrospective chart review	Africa, Eastern Europe, Asia	Asylum seekers	Contraception, abortion, & family planning	To quantify induced abortion and teenage birth indicators for asylum seekers
Gurnah (2011)	USA	Qualitative; Thematic analysis	Somali Bantu	Resettled refugees	Sexual healthcare; Contraception, abortion, & family planning	Explore the reproductive health experiences of Somali Bantu women in Connecticut to identify potential barriers to care experienced by marginalized populations
Hawkey (2021)	Canada and Australia	Qualitative, Thematic analysis	Bhutanese	Resettled refugees	Cervical cancer screening	To explore migrant and refugee women’s preferences for the delivery of SRH information and care
Haworth (2014)	USA	Mixed-methods; Likert scale survey and focus group questions based on Health Belief Model	Bhutanese	Resettled refugees	Cervical cancer screening & prevention	To investigate cervical cancer and screening knowledge and perceptions about the susceptibility and severity of cervical cancer and perceived benefits and barriers to screening
Henry (2022)	Australia	Qualitative; thematic data coding techniques	Not described	Resettled refugees; Fronline staff	Gender-based violence	To investigate the experiences and impacts of technology facilitatied domestic violence among immigrant and refugee women in Australia, and to understand their help-seeking pathways and sources of support
Inci (2020)	Germany	Quantitative; Cross-sectional survey	Syrian, Afghanistan, Iraq, Iran, Albania, Egypt, Kosovo	Resettled refugees	Contraception, abortion, & family planning	To describe the current reproductive health status of female refugees and to provide an initial overview of their existing unmet family planning and contraception needs
Kaneoka (2020)	Scotland	Qualitative; Interpretive phenomenology	Asia, Middle East, Africa	Refugees and asylum seekers	SRH education	To explore the SRHL-related views and experiences of adult ASRW living in Glasgow and their views on assistance required to improve their SRHL
Katcher (2021)	USA	Quantitative; survey with descriptive analyses	N/A	Healthcare providers or resettlement workers	SRH education	To examine the capacity and interest of resettlement offices in providing SRH information and referrals to newly arrived refugees
Keller (2007)	USA	Qualitative; Individual interviews (no methodology listed)	Sudanese	Healthcare providers	Gender-based violence	To understand what challenges Sudanese women face when accessing services for domestic violence
Kenny (2021)	USA	Quantitative; Retrospective chart review	Burmese	Resettled Refugees	Cervical cancer screening & prevention	To assess the vaccination and Pap smear rate among Burmese refugees in Omaha, Nebraska within one hospital system to inform future quality initiatives
Kim (2017)	South Korea	Mixed-methods; Survey; Individual interviews	North Korean	Resettled refugees	Cervical cancer screening & prevention	To examine the extent and pattern of health care utilisation among North Korean refugees before and after arrival to South Korea
Kue (2017)	USA	Quantitative; Survey	Bhutanese-Nepali	Resettled refugees	Cervical cancer screening & prevention	To understand rates of cervical cancer screening, barriers, facilitators, and beliefs about screening, and post-migration challenges
Kulig (1995)	USA	Qualitative; Ethnography	Cambodian	Resettled refugees	Cervical cancer screening & prevention	To understand men’s and women’s knowledge and use of family planning methods
Kumar (2021)	USA	Qualitative; Thematic text analysis and narrative approach	Burma, Central African Republic, the Democratic Republic of Congo, and Somalia	Resettled refugees	SRH education	To identify the facilitators and barriers to sexual health among refugee young women across multiple contexts
Kurth (2010)	Switzerland	Mixed-methods; Retrospective chart review; semi-structured interviews	50% from Yugoslavia, 19% Africa, 16% Asia, 9% from Eastern Europe, 6% other countries	Refugee-claimants; Healthcare providers	Contraception, abortion, & family planning	To identify reproductive health issues in a population of women seeking asylum in Switzerland, and to examine the care they received
Lor (2018)	USA	Qualitative; No explicit methodology	Burmese, Bhutanese	Resettled refugees	Cervical cancer screening & prevention	To understand the factors contributing to Burmese and Bhutanese refugee womens’ decisions about cervical cancer screening
Mehta (2018)	USA	Qualitative; Grounded Theory	Congolese, Somali	Resettled refugees and refugee-claimants	SRH education	To understand perspectives on gynaecological care for resettled Somali and Congolese refugees and asylum seekers
Mengesha (2017)	Australia	Qualitative; Thematic analysis, semi-structured interviews	N/A	Healthcare providers	SRH education; Contraception, abortion, & family planning Cervical cancer screening & prevention	To examine the perspectives and practices of Australian HCPs about the provision of SRH care for refugee and migrant women
Mengesha (2018)	Australia	Quantitative; Q-methodology	N/A	Healthcare providers	SRH education; Cervical cancer screening & prevention; Contraception, abortion, & family planning	To examine health care professional perspectives regarding the challenges of providing sexual and reproductive health care to refugee and migrant women using Q methodology
Metusela (2017)	Australia; Canada	Qualitative; thematic analysis, social constructivist epistemology	Afghanistan, Iraq, South America, Somalia, Sudan, India	Resettled refugees	SRH education	To determine how migrant and refugee womens’ constructions and experiences of SRH influence SRH behaviour
Ngum Chi Watts (2014)	Australia	Qualitative; Phenomenology with cultural competency framework & intersectional lens	Sudan, Liberia, Ethiopia, Burundi, and Sierra Leone	Resettled refugees	Contraception, abortion, and family planning; SRH education	To discuss the contraceptive knowledge and attitudes of teenagers and women of African descent who have experienced teen pregnancy, including the role of myths and misinformation
Ngum Chi Watts (2015)	Australia	Qualitative; Phenomenology with cultural competency framework & intersectional lens	Sudan, Liberia, Ethiopia, Burundi, and Sierra Leone	Resettled refugees	Contraception, abortion, & family planning	To examine contraception awareness and use among African Australian women, who have experienced teen-age pregnancy, and to explore the social contexts that shape these womens’ attitudes towards contraception
Njie-Carr (2021)	USA	Qualitative; Grounded Theory	Africa, Asian, South America	Resettled refugees	Gender-based violence	The objective of this analysis was to increase our understanding of immigrant and refugee women’s responses to abuse
Parajuli (2019)	Australia	Qualitative; Phenomenology	Bhutanese	Resettled refugees	Cervical cancer screening & prevention	To explore what a refugee women’s health screening program would look like if the views of Bhutanese refugee women were incorporated into service design
Parajuli (2020)	Australia	Qualitative; Interpretive Phenomenolog-ical Analysis	Bhutanese	Resettled Refugees	Cervical cancer screening & prevention	To identify perceived barriers to accessing cervical cancer screening programs among Bhutanese refugee women
Raben (2018)	Netherlands	Quantitative; Retrospective chart review	Three main countries of origin were Somalia (11.5%), Iraq (8.7%) and Nigeria (8.7%)	Refugee-claimants	Contraception, abortion, & family planning	To get insight into GP care related to contraception in refugees and other migrants compared with native Dutch women
Raines Milenkov (2020)	USA	Cross-sectional survey	Myanmar, Central Africa, Bhutan, Somalia, Arabic Speaking Countries	Resettled refugees	Cervical cancer screening & prevention	To assess differences in uptake of cervical, breast, liver, and colorectal screens across six cultural groups
Redwood-Campbell (2008)	Canada	Mixed-methods; Survey; Focus groups	Kosovo	Resettled refugees; Healthcare providers	Contraception, abortion, & family planning; Cervical cancer screening & prevention	To describe women’s reproductive and mental health-related issues after resettlement
Rees (2007)	Australia	Qualitative, Intersectional Feminism	Iraq, Ethiopia, Sudan, Serbia, Bosnia, Croatia	Resettled refugees	Gender-based violence	To examine the significance and interrelatedness of cultural, psychosocial and economic factors in the safety and wellbeing of refugee families experienc- ing domestic violence
Rodella Sapia (2020)	Switzerland	Qualitative; Framework Analysis	N/A	Healthcare providers and resettlement workers	Gender-based violence	This study explored the context GBV victims face when they seek refuge in Switzerland
Rogers (2014)	Australia	Qualitative; Psychosocial framework	Eritrean	Healthcare providers	Contraception, abortion, & family planning	To explore and document Sudanese and Eritrean womens’ intergenerational experiences and knowledge of reproductive health and contraception
Rogers (2015)	Australia	Qualitative; Psychosocial framework	Eritrean	Resettled refugees	SRH education	To investigate and document the experiences and knowledge of sexual and reproductive health and contraception and views on sexuality and sexual relationships among Sudanese and Eritrean women
Rogstad (2004)	United Kingdom	Quantitative, Chart review	Not listed	Refugee-claimant	Cervical cancer screening & prevention	This study aimed to discover the needs of asylum seekers attending a sexual health clinic and if they are significantly different from those of British-born patients
Rubens-Augustson (2019)	Canada	Qualitative; Content analysis	NA	Healthcare providers	Cervical cancer screening & prevention	To understand, from the perspective of healthcare providers, barriers and facilitators to HPV vaccination, and recommendations to improve HPV vaccine uptake among newcomers
Russo (2019)	Australia	Qualitative; Community-Based Participatory Research	Afghani	Resettled refugees	Contraception, abortion, & family planning	To explore the family planning perspectives and experiences of Afghan people living in Australia
Schuster (2019)	USA	Qualitative; Community-Based Participatory Research	Somali Bantu, Karen	Resettled refugees	Cervical cancer screening & prevention	To characterize Somali Bantu and Karen experiences with cancer and cancer screenings prior to and after resettlement in Buffalo, NY in order to inform engagement by health providers
Sheeran (2022)	Australia	Qualitative; reflexive thematic analysis	NA	Service directors (lawyers, counsellors, social workers)	Gender-based violence	To understand what role language plays in help-seeking for migrant and refugee women experiencing domestic and family violence that may include reproductive coercion and abuse/sexual violence
Soin (2020)	USA	Qualitative, Grounded Theory	Ethiopian	Resettled refugees	Gender-based violence	To understand how to effectively counsel and provide culturally sensitive family planning care to resettled refugee women
Sullivan (2005)	USA	Qualitative; Participatory Action Research	Ethiopian	Resettled refugees	Gender-based violence	To understand the experiences of domestic violence among Ethiopian refugee women in the USA
Svensson (2017)	Sweden	Qualitative; Content Analysis	Afghani, Iraqi, Somali, Iranian	Resettled refugees	SRH education	To gain an understanding of how education concerning SRH and rights was perceived by participants
Valdovinos (2021)	USA	Qualitative; Narrative Analysis	Mexico, El Salvador, Guatemala	Refugee-claimants	Gender-based violence	To explore Latina immigrant women’s experiences of IPV by using an intersectional Chicana feminist approach
Vangen (2008)	Norway	Quantitative; chart review	Eritrea, Somalia, Iraq, Iran, Sri Lanka, Vietnam, Chile, Serbia, Bosnia	Resettled refugees	Contraception, abortion, & family planning	To evaluate the termination of pregnancy rates and risk factors related to immigration status
Wachter (2019)	USA	Qualitative; Thematic Approach	South/South-East Asia, Central Asia, West and North Africa, Middle East, Central America	Resettled refugees; Service providers	Gender-based violence	To develop a more nuanced understanding of domestic violence needs as expressed by women resettling to the United States as refugees.
Wachter (2021)	USA	Qualitative; Interpretive thematic approach	Mexico, El Salvador, Guatemala	Resettled refugees	Gender-based violence	To examine factors that hinder help-seeking for intimate partner violence among women who resettled to the United States as refugees
Wachter (2022)	USA	Qualitative; thematic approach	NA	Social service providers	Gender-based violence	To examine organizational factors infuencing the availability and accessibility of IPV services for refugee and other vulnerable immigrant women in the U.S. from the perspectives of social service providers
Wiedmeyer (2012)	Canada	Quantitative; Chart review	90% non-European country of origin, 10% European country of origin	Resettled refugees	Cervical cancer screening and vaccination	To see if refugee women at a community health centre (CHC) in Toronto, Ont, are appropriately screened for cervical cancer and if there are any demographic characteristics that affect whether they are screened
Zannettino (2012)	Australia	Qualitative; Nested Ecological Model	Liberian	Resettled refugees	Gender-based violence	To explore the factors that have an impact on domestic violence in African refugee communities
Zhang (2017)	USA	Qualitative; in-depth interviews	N/A	HCPs	Cervical cancer screening & prevention	To identify health and social service providers’ perspectives on promoting cervical cancer screening in order to inform the development of effective programs to increase screening among recently resettled refugees
Zhang (2020)	USA	Qualitative; Grounded Theory Methodology	Somali	Resettled refugees	Contraception, abortion, & family planning	To explore the knowledge, attitudes, and experiences of Somali refugee women with family planning in the US

Our findings are reported in two sections, the first detailing use of SRH services (objective 1) and the second to identify barriers and facilitators influencing access to SRH care (objective 2) among refugee and asylum-seeking women in high-income countries. Each of these two sections have been presented within four overarching categories: gender-based violence, family planning, cervical cancer screening and prevention, and SRH education, to offer a more detailed understanding of factors shaping access to different types of services. These services and supports were most frequently discussed across all included studies. The majority of papers focused on contraception and family planning (n = 23; [13, 23, 40, 48, 54, 59, 66, 72–74, 78–80, 85, 86, 89, 91, 95, 96, 99–101, 103] followed by cervical cancer screening (n = 20; [41, 42, 47, 49, 52, 53, 56, 58, 59, 65, 69, 70, 72, 73, 75, 76, 86–88, 102] gender-based violence (n = 17; [43, 44, 46, 51, 61–64, 67, 68, 77, 81–84, 90, 93] and SRH education (n = 12; [12, 13, 33, 45, 50, 55, 71, 73, 79, 92, 94, 98]. Given the large volume of data reported in objective 2, the Health Behaviour Model [31] was utilized to further organize the data.

### Use of sexual health services

The first objective of this study was to understand the *use* of sexual health services among refugee and asylum-seeking women in high-income countries. The results of this section are stratified by most discussed service type (i.e., GBV; family planning, contraception, and abortion; cervical cancer screening; sexual health education). The majority of studies documenting use of SRH services were quantitative. Quantitative summary statistics are reported in Table 2.

**Table 2:** Quantitative data from included studies relating to *use* of sexual and reproductive healthcare (SRH) among refugee and asylum seeking women in high-income countries, n = 61.

Study	Country of resettlement	Study ethnicity or country of origin	Topic	Main finding
Agbemenu 2020	USA	Africa (DR Congo), Somalia, Kenya	Contraception	35% of refugee women in the sample were currently engaging in family planning
				Injectable: 13%; Condom 7%; IUD or implant: 6%; Pills: 5%; Sterilization 2%; Natural methods 1%
Allen 2019	USA	Somali	Cervical cancer screening	74.1% of refugee women in the sample had received a pap smear in the past three years
Aptekman 2017	Canada	63.5% European, 13.5% African, 11.5% Asian, 11.5 South/Latin American	Family planning	69.2% of refugee women in the sample were currently engaging in family planning
				IUD: 7.7%; Implant: 1.9%; Condoms: 23.1%; Sterilization: 15.4%; Withdrawal: 3.8%; Unknown: 17.3%
Barnes 2004	USA	31% Cuban, 26% Bosnian, 25% Vietnamese, and 18% other ethnicities	Cervical cancer screening	24% of refugee women in the sample had received a pap smear in the past three years.
Dean 2017	Australia	Sudanese	Family planning	13.6% of refugee youth in Australia had reported at least one abortion in their lifetime
Degni 2006	Finland	Somali	Family planning	27% of refugee women in the sample were currently engaging in family planning
				IUD: 18.0%; Pills: 7.0%; Injectables: 2.0%
Goosen 2009	The Netherlands	African, Asian	Family planning	Overall abortion rate among asylum-seeking women was one-and-a-half times higher than women born in the Netherlands (8.6/1000 per year; 14.4/1000 per year)
Haworth 2014	Canada & Australia	Bhutanese	Cervical cancer screening	Refugee women sampled reported a 62.5% lifetime engagement in cervical cancer screening
Inci 2020	Germany	Syrian, Afghani, Iraqi, Iranian, Albanian, Egyptian	Family planning	31.6% of refugee and asylum-seeking women were currently engaging in family planning
				Withdrawal: 10.7%; IUD: 9.4%; Condoms: 3.9%; Pills: 2.9%; Calendar: 2.6%; Sterilization: 1.3% Injectables: 0.3%
Kenny 2021	USA	Burmese	Cervical cancer screening	32% of refugee women had received a pap smear in the past three years.
Kue 2017	USA	Bhutanese-Nepali	Cervical cancer screening	44.3% of refugee women reported lifetime engagement with cervical cancer screening
Kulig 1995	USA	Cambodian	Family planning	13.2% of participants were using contraception at the time of study
Kurth 2010	Switzerland	50% Yugoslavian, 19% African, 16% Asian, 9% Eastern European, 6% other ethnicities	Family planning	69% of asylum-seeking women were engaging in family planning.
				Pills: 21.0%; IUD: 27.0%
Lor 2018	USA	Burmese, Bhutanese	Cervical cancer screening	55% of refugee women had a pap smear since arriving in the US
Parajuli 2019	Australia	Bhutanese	Cervical cancer screening	50% of refugee women had a pap smear since arrival
Raben 2018	The Netherlands	The women originated from 52 different countries. Three main countries of origin were Somalia (11.5%), Iraq (8.7%) and Nigeria (8.7%)	Family planning	Significantly higher rate of abortion service use among refugee women (11.5%) compared to Dutch women (3.7%, *p* < 0.001)
				33.7% of refugee women sampled were actively using family planning. Pills: 22.0%; IUD: 7.0%; Injectables: 4.0%
Raines-Milenkov 2020	USA	Myanmar, Central Africa, Bhutan, Somalia, Arabic Speaking Countries, and others	Cervical cancer screening	37% of refugee women had received a pap smear in the past three years
Redwood-Campbell 2008	Canada	Kosovo	Family planning	14.3% engagement in family planning
Rogstad 2004	UK	Not described	Cervical cancer screening	27.8% of asylum-seeking women had a pap smear since arrival in the UK
Schuster 2019	USA	Somali Bantu, Karen	Cervical cancer screening	67% of refugee women had a pap smear since arrival in the US. No participants had been tested before resettlement
Vangen 2008	Norway	Mixed (Eritrea, Somalia, Iraq, Iran, Sri Lanka, Vietnam, Chile, Serbia, Bosnia)	Family planning	Refugee women in Oslo, Norway had significantly higher rates of abortion when compared to Norwegian-born women (AOR 1.94, 95% CI 1.79–2.11)
Wiedmeyer 2012	Canada	90% non-European country of origin, 10% European country of origin	Cervical cancer screening	284 of the 357 eligible charts (80%) contained at least 1 Pap test result within the study period. The average length of time to first Pap test was 140 days for those women who had one.

#### Use of family planning services

Nine studies reported on use of contraception or engagement in family planning (25,40,82,89,97–99). The use of family planning methods (inclusive of natural, hormonal, long-acting, and permanent methods) among refugee and asylum-seeking women varied widely across studies, ranging from 13.2% to 69.2%. In North America (Canada, USA) rates of engagement ranged from 13.2% to 69.2%. In Europe, it ranged from 27% to 69%. Condoms remained the most often used method, followed by intrauterine devices, natural methods, and oral contraceptives. Two studies reported on the unmet need for contraception among asylum-seeking and refugee women. This was calculated by measuring the proportion of women who were not trying to conceive and who were not engaging in an effective method of family planning/pregnancy prevention. Unmet need for contraception was high, ranging from 26.8% (Canada; 85) to 47% (Germany; 21), both of which were significantly higher than the host population.

Eight studies reported on the use of abortion services among refugee and asylum-seeking women [23, 54, 71, 86, 91, 95, 96, 101]. All studies reported a significantly higher rate of abortion use across refugee and asylum-seeking women, when compared to women born in the host country, ranging from 1.5 to. Goosen et al. (2009) reported that the abortion rate among refugee women was 1.5 times higher than women born in the Netherlands [95]. Similarly, Raben et al. (2018) reported a significantly higher rate of abortion service use among refugee women (11.5%) compared to Dutch women (3.7%, *p* < 0.001). Kurth et al. (2010) reported that induced abortions were the most performed procedure among asylum-seeking women surveyed; 22.5% of participants had received an abortion since arriving in Basel, Switzerland. In Oslo, Norway, refugee women had significantly higher rates of abortion when compared to Norwegian-born women (AOR 1.94, 95% CI 1.79–2.11) [101].

#### Use of cervical cancer screening and preventative care

Twelve studies reported on cervical cancer screening (pap smears) and HPV vaccination [41, 42, 49, 52, 53, 56, 58, 59, 76, 86, 88, 97]. Limited information was available on rates of HPV uptake, with only two studies reporting on this outcome. Kenney et al. (2021) reported that 49.2% initiated and 30.8% of Burmese refugee women completed their HPV schedule in Omaha, Nebraska [52]. Allen et al. (2019) reported that 23% of Somali refugee women had an HPV vaccine and 58% had their children vaccinated in Minneapolis. The rate of cervical cancer screening varied widely (see Table 2). Across the US, rates of engagement with cervical cancer screening ranged from 18% to 74.1%, predominantly among Burmese and Bhutanese refugee women [41, 42, 49, 52, 53, 56, 58, 59]. Among Canada, the United Kingdom, and Australia rates ranged from 25.9% to 80% among a range of multiple ethnicities and countries of origin. [76, 86, 88, 97]

#### Gender-based violence service use

Ten studies discussed use of services related to gender-based violence (seven quantitative; one qualitative; [43, 44, 51, 61, 63, 67, 77, 81, 83, 93]. Women utilized a variety of services/supports for gender-based violence, including social services [61, 83], legal supports [61], economic assistance [61], law enforcement [44, 61, 83], shelters [61, 63, 67], and psychological or emotional counselling [63, 67, 83, 97].

#### Sociocultural and structural factors influencing access to sexual health services

**Fig 2:**
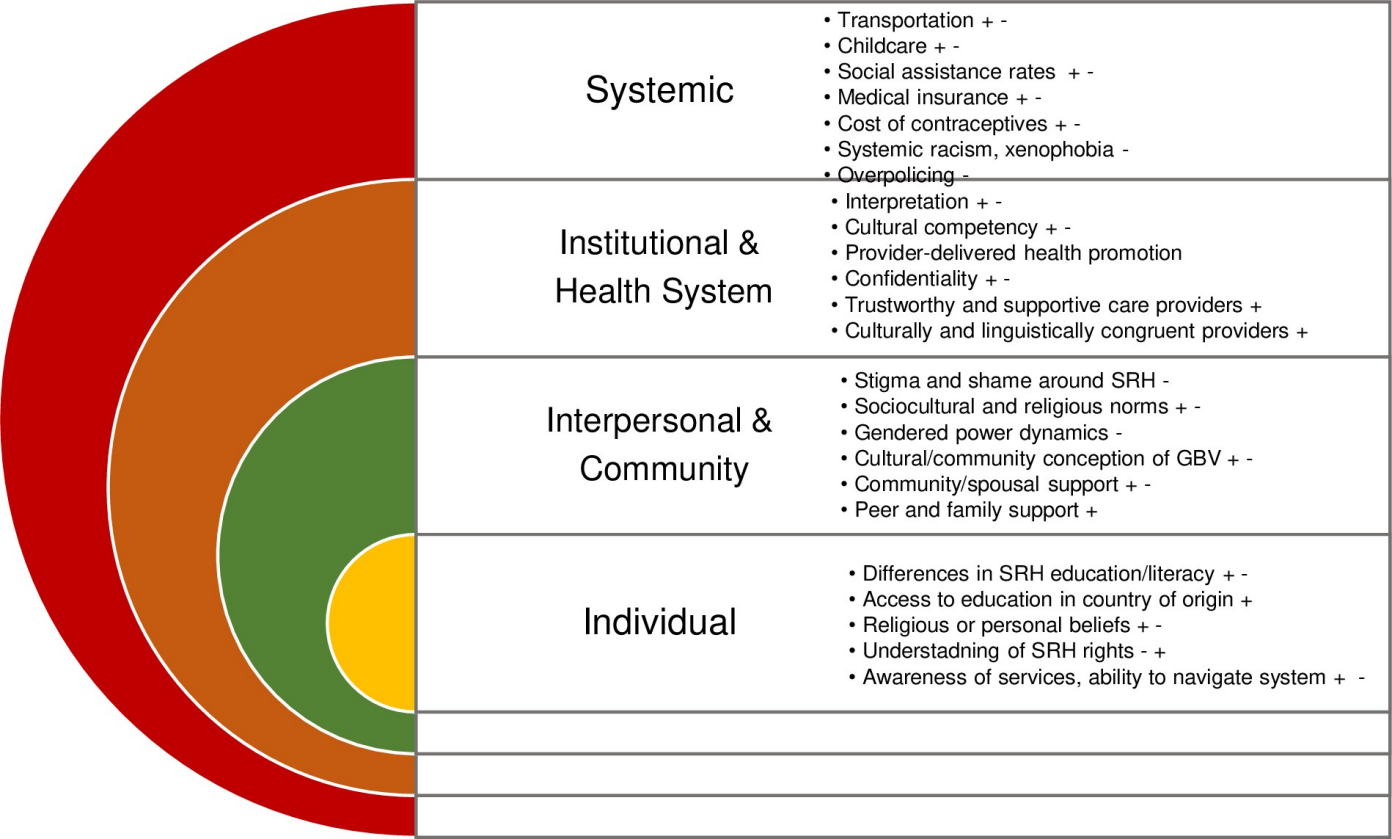
Barriers and facilitators influencing access to services, stratified by individual, community, institutional and systemic factors (modeled after Tirado et al. (2020);—indicates barriers, + facilitators).

This section addresses review objective 2: to understand the barriers and facilitators influencing *access* to sexual healthcare for refugee and asylum seeking women in high-income countries. The results of this section are stratified by most discussed service type (i.e., GBV; family planning, contraception, and abortion; cervical cancer screening; sexual health education). Each section is then divided into the four facets of the Health Behaviour Model [31]. Fig 2 details prominent factors influencing access to sexual health services stratified across the four levels of the Health Behaviour Model. Table 3 details important, relevant quotations across strata. Tables 4 and 5 provide a summary or barriers and facilitators described across included studies.

**Table 3:** Sample quotes from included qualitative articles relating to access to sexual and reproductive healthcare (SRH) among refugee and asylum seeking women in high-income countries, n = 61.

Facet of the Health Behaviour Model and categories of SRH	Example quotes from refugee or asylum-seeking women, stakeholders, or healthcare providers
Individual	
GBV	“In their country [or origin], if you see a psychologist, you have a problem in your head. That’s why they don’t want to seek psychological support. That’s very stigmatizing…” Stakeholder, Switzerland [90]
Family planning	“The only thing I know is about using condoms. So, for me when I talk about safety, it means that to use condoms while having sex so that you will not get pregnant.” Bhutanese refugee woman, USA [45]
	At the time, I didn’t know anything at all … when I came [to Australia] I had my son [fifth child] and he was about nine months, and we went to the doctor and the doctor asked me if I’m on the pill and I said I don’t know what that pill means, and she explained it to me. I told her I never heard about something like that, and she said, ‘If you don’t do something about yourself you’re going to get yourself pregnant.’” African refugee woman, Australia [13]
Cervical cancer	“They’re not aware that we have those [screening] services available, especially if those types of services were not available in their own country” Healthcare provider, Australia [73]
SRH Education	I don’t know much about how to prevent myself getting it [STI]…what kind of problems or infections there are, but I try to keep myself very clean.” Afghani refugee woman, Canada [12]
Community	
GBV	“People are finding themselves ostracized from their support network because they are taking a stand against the violence in the house. A lot of our victims have to wrestle with that in terms of moving forward and continuing on and staying out of the abusive relationship.” Stakeholder, US [44]
	“In [our home country] women have family to defend them, here they know they have no one to defend them, they beat them, they are at risk here” Refugee woman, Australia [77]
Family planning	“In Somalia, a woman could never discuss the number of children with her husband, or the use of contraception because of the Somali family traditions and religious teachings, but in Finland, Somali women do discuss family matters with their husbands, including the sensitive issue of birth control.” Somali refugee woman, Finland [99]
	“I remember doing some sessions with women about contraception and they actually knew a lot of stuff. Then I said ‘so you know all this stuff, but you’re not using contraception. Why not?’ They said ‘because it’s not our choice to make. It’s our husband’s choice.’” Stakeholder, Australia [73]
	“I think our girls, they are not doing abortion, because it’s not culturally accepted.” Bhutanese refugee woman, USA [45]
Cervical cancer	“I think there is no problem for me or the women of my age, but our mothers are aged women and are very reluctant to do this test until, and unless, it is requested by a doctor or they have problems related to this. They are very shy, and they have a deeply rooted concept that this part [of their body] is not to be seen by anyone except their husband.” Refugee woman [76]
SRH Education	“All of the information that I had about getting married and sex was from books, but not from my family or my mum, because she was shy to tell me anything about it.” Sudanese refugee woman, Canada [12]
	“I didn’t know that I will become pregnant the first time I started bleeding. Our parents were not educated. They didn’t know how to communicate with their children.” Somali refugee woman, Canada [12]
Institutional	
GBV	“Even if we know what resources are there, we don’t know how to go about it, where to start. So, if my friend decides she going to leave her husband, and she asks me, I would refer to somebody who might know this kind of information. Maybe someone who has been here longer. Because if I don’t know, I can’t help her… Everything is so hard, just getting things done.” Refugee woman, US [64]
Family planning	“Wherever we go…they [Israeli healthcare providers] don’t listen to us… They call us ‘kushi’ [pejorative term for dark-skinned person]…and their impression is that we are worthless.” Eritrean asylum-seeking woman, Israel [100]
	“Almost all young women who transited through Egypt have an IUD. I can recognize that it is not from here because the thread is different. They have all been to a clinic in Egypt. Some of them know, but many of them don’t know about the IUD.” Healthcare provider, Switzerland [90]
Cervical cancer	“Personally, though I’m ok with my GP but only for general health and wellbeing because when it comes to female matters I would rather see a woman professional not a man…All my women’s health issues have been done by a female practitioner.” Sub-Saharan refugee woman, Australia [69]
	“It’s very, very hard, because we don’t understand the language. That’s why it’s so hard. The clinic always has to call an interpreter for me. I have to say that I am lucky if I get a kind and patient interpreter.” Burmese refugee woman, USA [56]
SRH Education	“Culture competency of service providers and educators is an essential component for culturally sensitive sexual and reproductive education and sexual and reproductive health knowledge to be shared. Health care information accessible for people from non-English speaking backgrounds with varying degrees of literacy is fundamental in increasing SRH knowledge within minority ethnic communities. Stakeholder, Australia [79]
Systemic	
GBV	“One day [husband] walked in and found her packing and became furious, pulled out a gun, and to threatened to kill himself if she left… The police arrived, but were unable to grasp the critical nature of the situation and did not take them seriously. They made no attempt to contact the language line [on-demand phone interpreting service]. Instead, the officer began to question why they were not using a car seat for the baby. Through their persistence, they were able to get the message across and persuaded the officers to go the apartment.” Stakeholder, USA [44]
Family planning	“Where do they find money to buy an IUD?. . . Some groups of women did not have public health [Medicare] benefits… The waiting lists for Medicare based appointments are unreasonable at times” Healthcare provider, Australia [73]
Cervical cancer	“If we have health insurance from our spouse’s work, we have to pay a co- payment. Some are not covered for every visit, and so sometimes we hesitate to see our doctor because we don’t have money to pay for our co-payment. We want to visit a clinic, but we don’t have enough money.” Burmese refugee woman, USA [56]
SRH Education	“Well, they really have other burning issues to address. So their burning issues are housing, where to live with their family, how to live, and the schools for kids, learning the language, employment. All these things are more prioritized that sexual reproductive health.” Stakeholder, Australia [73]

**Table 4:** General barriers to sexual and reproductive health services collapsed across sub-categories.

Study ID	Language/ commun-ication differences	Differences in SRH literacy	Stigma and shame	Service cost	Health system navigation	Lack of cultural competency of providers	Unable to prioritize care	Interpreter access	Misinformation/ myths	Male providers	Religious restrictions	Lack of transportation	Discomfort with interpreters	Discrimination, xenophobia or racism
**Total**	34	31	31	20	17	16	15	15	13	11	10	8	6	5
**Agbemenu 2020**		X							X					
**Allen 2019**		X							X					
**Babatunde-Sowole 2020**		X		X			X		X	X				
**Bartlett 2020**	X	X		X	X			X				X		
**Byrskog 2015**	X					X								
**Critelli 2019**	X	X	X			X	X	X						
**Degni 2006**						X								
**De Anstiss 2023**	X		X		X									
**Dhar 2017**	X	X	X			X			X					
**Fineran 2020**	X		X	X										
**Gebreyesus 2020**	X		X	X		X	X	X				X		X
**Ghebre 2015**	X	X	X	X	X		X		X	X	X		X	
**Gurnah 2011**	X		X	X	X	X		X		X	X			
**Hawkey 2021**		X	X				X			X				
**Haworth 2014**	X	X	X	X	X			X	X	X		X	X	
**Henry 2022**	X				X	X								
**Kaneoka 2020**	X	X	X		X			X		X	X			
**Inci 2020**		X												
**Katcher 2021**	X	X	X				X							
**Keller 2007**	X	X	X		X	X		X						
**Kim 2017**		X		X			X		X	X				
**Kue 2017**	X		X				X							X
**Kulig 1995**	X													
**Kumar 2021**			X								X			
**Kurth 2010**	X			X				X						
**Lor 2018**	X	X	X	X	X		X	X	X			X		
**Mehta 2018**		X	X	X		X				X	X	X		
**Mengesha 2017**	X	X	X	X	X		X							
**Mengesha 2018**	X	X		X	X	X					X		X	
**Metusela 2017**		X	X											
**Ngum Chi Watts 2015**		X	X				X		X					
**Ngum Chi Watts 2014**		X	X						X					
**Parajuli 2020**	X	X	X			X	X	X						
**Parajuli 2019**		X												
**Redwood-Campbell 2008**	X			X				X				X		
**Rees 2007**			X											X
**Rodella Sapia 2020**	X		X			X		X					X	X
**Rogers 2014**	X	X	X	X		X	X		X	X	X			
**Rogers 2015**		X	X			X								
**Rubens-Augustson 2019**	X	X	X	X	X		X				X			
**Russo 2020**	X		X	X						X	X		X	
**Schuster 2019**	X	X		X								X		
**Sheeran 2023**				X	X			X						
**Soin 2020**	X	X	X										X	
**Sullivan 2005**	X	X			X							X		
**Svensson 2017**	X	X	X		X				X	X				
**Valdovinos 2021**			X	X		X								X
**Wachter 2019**		X		X	X									
**Wachter 2022**	X			X		X		X						
**Wiedmeyer 2012**	X													
**Zannettino 2012**			X											
**Zhang 2017**	X				X		X	X						
**Zhang 2020**			X						X		X			

**Table 5:** General facilitators to sexual and reproductive health services collapsed across sub-categories.

Study ID	Support from care provider	Community programming/ workshops	Comfort with providers	Education/materials in languages	Interpreters	Education from care provider	Supportive family	Health insurance or financial aid	Education on social media	Female provider	Multilingual providers	Cultural competency training	Web-based info	Supportive partner	Recommendations from family or friends	Education for men
Frequency	**16**	**14**	**12**	**11**	**11**	**9**	**9**	**8**	**6**	**7**	**7**	**6**	**4**	**4**	**4**	**4**
**Allen 2019**	X	X				X	X		X					X		
**Babatunde-Sowole 2020**										X						
**Bartlett 2020**							X									
**Byrskog 2015**	X		X								X					
**Critelli 2019**	X	X	X								X	X				
**Fineran 2020**		X						X				X				
**Gurnah 2011**			X		X			X				X				
**Hawkey 2021**	X	X	X			X										X
**Haworth 2014**				X				X								
**Kaneoka 2020**		X		X	X	X			X				X			
**Keller 2007**			X													
**Kim 2017**							X	X								
**Kue 2017**	X						X								X	
**Kumar 2021**	X	X	X	X			X		X							
**Kurth 2010**					X			X		X						
**Lor 2018**	X		X				X									
**Mehta 2018**										X						
**Mengesha 2017**				X	X										X	
**Mengesha 2018**											X	X				
**Njie-Carr 2021**					X		X	X		X	X					
**Parajuli 2019**	X				X	X									X	
**Parajuli 2020**	X					X										
**Rodella Sapia 2020**	X		X												X	
**Rogers 2014**		X		X	X		X				X			X		X
**Rogers 2015**	X		X				X			X	X	X				X
**Rubens-Augustson 2019**	X		X	X		X		X								
**Russo 2020**			X	X	X	X							X	X		
**Schuster 2019**	X	X		X	X	X		X	X	X			X	X		
**Soin 2020**	X	X	X													
**Sullivan 2005**		X		X					X							
**Svensson 2017**	X	X		X	X				X	X			X			
**Wachter 2019**		X														
**Wachter 2022**											X	X				
**Zannettino 2012**		X														X
**Zhang 2017**	X	X		X	X	X										

#### Gender-based violence

*Individual*. Four studies reported refugee and asylum-seeking women often have a limited knowledge of their host countries legal system or were unclear about their personal rights and protections [44, 51, 61, 63]. This left women unaware of where to seek help or what services were available to them. Four studies described women holding mixed perceptions around what constitutes intimate partner violence [44, 61, 62, 93]. For example, Byrskog et al. (2015) described Somali-born women as unfamiliar with certain forms of abuse, particularly psychological and financial abuse, and did not consider non-consensual sex to be a form of violence. Two studies indicated a need for linguistically/culturally appropriate programs to educate women on their personal rights, what constitutes domestic violence, and how to navigate the legal system [44, 61].

*Community*. Six studies reported that refugee and asylum-seeking women often did not have the appropriate amount of social and emotional support to seek help, as they had been isolated from friends and family through forced displacement and international resettlement [44, 51, 61, 62, 77, 84]. This limited women’s ability to leave abusive situations and identify and navigate services [44, 51, 61, 62, 77]. Other women were deterred from seeking support or taking legal action owing to a fear of being stigmatized within their community, particularly if they pursued separation or divorce, or had concerns around encountering victim-blaming [44, 46, 61–63, 81, 83, 90]. Other studies of Ethiopian and Somali refugee women reported acceptance and normalization of domestic violence among some members of their community [61, 93]. This normalization pressured women to remain silent, fearing a lack of support from community services, family, or friends. Refugee women felt pressured by their community and/or extended family to avoid seeking help, feeling concern around how it may impact their perception [44, 61, 77, 81]. Three studies indicated that women wanted intervention and education supports for their partners and/or other men in their communities [63, 81, 92]. For those who did have the support of community, family, or friends, this was a facilitaror for accessing and navigating GBV-related services [68]

*Institutional*. Eight studies detailed concerns related to interpretation [44, 51, 67, 82–84, 90, 93], including a lack of interpretation services and discomfort with the use of male interpreters when disclosing GBV [51, 93]. Fineran et al. (2020) noted that male partners may be more proficient in English and may use that to gate-keep women from English-only services. Women who had used shelter services reported encountering racism and xenophobia from other service users and employees [44, 62, 93]. Likewise, Wachter et al (2022) reported that GBV services were not equipped to care for refugee populations, with staff no reflecting the demograpics of their clients. Moreover, service providers felt ill-equipped to respond to the mental healthcare needs of refugee clients who had experienced multiuple traumas. This only perpetuates additional violence and may force women back to their abuser. Other studies described how challenging it can for refugees to naviagate services, especially regarding the reporting of violence, which is compounded by linguistic differences [82–84]. Byrskog et al. (2015) noted that perinatal healthcare appointments were critical opportunities for GBV screening, as there is often continuity of care, the opportunity to build safe, trusting relationships.

*Systemic*. Seven studies reported women—especially asylum-seekers—feeling fearful about reaching out to law-enforcement following an assault or incident of domestic violence, owing to their non-permanent status [44, 51, 61, 62, 81, 83, 90]. Women’s fear was multi-faceted. Four studies indicated that women did not trust police and were concerned that they may contact immigration authorities, which could impact their case to stay [44, 81, 90, 104]. Others reported that women were concerned that child protective services would remove their children if it was reported that there had been domestic violence in the home [44, 46, 62]. In some studies, women had gone to police seeking support and were not believed, not taken seriously or encountered racial or xenophobic discrimination [44, 62, 77]. Four studies also indicated that women were not financially independent and did not have the means to leave their partners [44, 46, 62, 63]. Other studies detailed the impact of partner criminalization, where women were apprehensive of reporting incidents of intimate partner violence, as it could have implications on their family income and financial stability [46, 81]. Wachter et al. (2019) and others outlined a critical need for financial assistance to give women the support to live alone, and outside of shelters [67, 83].

#### Family planning, contraception, and abortion

*Individual*. Four publications detailed misinformation around the mechanisms of hormonal contraception among women originating from a variety of African nations, including, Ethiopia, Sudan, Liberia, Burundi, DR Congo, Kenya, and Bhutan [13, 45, 66, 74]. The prevailing myth across studies was that the effects of hormonal contraceptives were non-reversable, and long-term use would result in permanent infertility, fetal abnormalities in future pregnancies, higher risk of miscarriage, or cause maternal diseases [13, 45, 74, 99]. 16.9% of participants in Agbemenu et al. (2020) reported fear of side effects influencing use of contraceptives [40]. Persistence of these misconceptions influenced use of contraceptives [13, 45, 66, 74, 99]. Ngum Chi Watts et al. (2014) noted that contraception was often not widely available or affordable in women’s countries of origin, and was often seen as a luxury for wealthier families, which contributed to a limited understanding of effective family planning upon arrival in resettlement countries.

#### Community

Sociocultural and religious norms influenced the uptake and use of modern contraceptives [13, 45, 66, 74, 78, 79]. Six studies reported that particular religious and ethnic groups valued and promoted abstinence before marriage [55, 78, 79, 92, 94, 98] This created stigma for sexually active, unmarried women who were seeking information around pregnancy prevention, limiting intergenerational and peer-to-peer knowledge sharing [13, 45, 60, 66]. Even for married women, cultural expectations and value placed around having large families—particularly among Somali women [66, 99]—restricted the acceptability and use of modern contraceptives [60]. This created additional shame and secrecy around accessing and using hormonal contraception for both married and unmarried women [13, 45, 66, 74, 78, 79]. Though several studies stated these ideals changed for many families upon arrival in high-income countries, as parents faced less social support, greater economic instability, and women had more educational and occupational opportunities outside the home [60, 66, 80, 99]. The persistence of patriarchal ideologies and power differences within relationships contributed to challenges negotiating the use of condoms [74, 80] and women’s husband not permitting or restricting the use of contraceptives [73, 80, 86, 99]. For example, four studies stated that women felt their religion did not condone the use of contraception or abortion—yet also stigmatized pregnancy in unmarried women [45, 55, 80, 99]. Supportive and open relationships with partners and families contributed to improved access to contraceptives and more open conversations around family planning [55, 78–80].

*Institutional*. Lack of interpretation provision was a critical barrier described across seven studies [48, 54, 73, 78, 86, 91, 100]. Three studies found that healthcare providers possessed stereotypes about Muslim women, assuming they would not be interested in hormonal contraceptives. As a result, some healthcare providers did not ask about it, due to this false assumption [48, 73, 96]. Indeed, Raben et al. (2018) found that physicians in the Netherlands discussed contraceptives significantly less often with refugees (51%) than with native Dutch women (84%; P < 0.001). Rodella Sapia et al. (2020) notes that a high proportion of asylum-seeking women who transitioned through Egypt had an intrauterine device inserted as part of a mandatory gynecological assessment and that many had this implanted without their knowledge or consent. Lor reported that some Bhutanese women had been assaulted by male healthcare providers in refugee camps and, as a result, were apprehensive about accepting care from Western, male healthcare providers [56]. These and other examples of forced or unsafe gynecologic care has resulted in feelings of mistrust among many resettled refugees and asylum-seekers [66, 90]. Culturally and linguistically congruent care providers are a key facilitator, removing the need for interpreters [72, 78, 79].

*Systemic*. Across a number of high-income countries including Switzerland, the United States, Israel, and Australia, women were required to pay in-part or in-full for any modern contraception, with higher up-front costs for long-acting contraceptives (e.g., intrauterine devices, sub-dermal implants; [48, 72, 73, 86, 91, 100]). Lack of coverage for contraception was linked to an increase in abortions in Switzerland [91].

### Access to cervical cancer screening and prevention

*Individual*. Six studies cited differences in health literacy as a barrier to cervical cancer screening and uptake of HPV vaccinations, owing to a lack of access to services and education in their country of origin, transit, or resettlement [41, 47, 49, 56, 69, 75]. Awareness and knowledge around cancer more broadly was high, but limited around ovarian and cervical cancers. For example, Haworth (2014) noted that only 22.2% of respondents had ever heard of a pap smear. Refugee and asylum-seeking women were often unfamiliar with the etiology of cervical cancer and contractability of HPV or had been misinformed about the development of the disease [41, 49, 56, 69]. Allen et al. (2019) found that Somali refugees surveyed had learned that cervical cancer was linked to a genetic predisposition and use of birth control.

A number of studies reported a general unfamiliarity with the concept and prioritization of preventative healthcare [47, 49, 53, 56, 65, 69, 73, 75, 102]. This was inexplicably linked to resource limitations and service costs in countries of origin and refugee camps, with many families forced to seek care only when disease or infection was clearly present [69]. Upon resettlement, many women may be unaware of the need for regular cervical swabs. Schuster et al. (2019) reported that 80–100% of Somali Bantu and Karen participants wanted more information about preventative cancer screening. Other studies reported that women who had experienced sexual or reproductive traumas may feel reluctant to have a pap smear, due to the invasiveness of the procedure [49, 56, 69]. Ghebre et al. (2015) further noted that women who had undergone female genital mutilation may have difficulties with the insertion of a testing swab.

*Community*. Three studies reported participants noting that cervical cancer screening would be inappropriate for unmarried women, due to the assertion that testing may imply the woman is sexually active [47, 57, 87]. Unmarried women were concerned that receiving a pap smear or HPV vaccination may indicate that they are sexually active and could be stigmatized by their family, peers, or community [47, 49, 57, 87]. For example, 57.1% of Bhutanese-Nepali women in the US indicated that ‘shyness’ prevents them from getting a pap smear. Some parents were concerned that having their children vaccinated against HPV may contribute to or promote pre-marital sex, which was deemed as inappropriate within their cultural beliefs/norms [47, 49, 57, 87]. Support and recommendations from peers and family members contributed to an increased uptake in cervical cancer screening [53, 73, 76].

*Institutional*. Poor or no interpretation contributed to lack of informed consent, inability to obtain consent, and poor procedural explanations, which left many women feeling confused, uncomfortable or even traumatized [59, 75, 87, 105]. Wiedmeyer et al. (2012) found that English-proficiency significantly predicted the likelihood of getting a pap smear after registration with a community health clinic (AOR 0.625, 95% CI 0.462–0.854). Healthcare providers have been reported to assume a lower level of health literacy among refugee women and have not properly explained the purpose of their procedures, violating principles of informed consent [59]. These and other experiences of discrimination or maltreatment led to reported feelings of mistrust among participants in two studies [47, 59]. However, women across other studies indicated that they had learned about cervical cancer, pap smears, and HPV and the need for vaccination from their healthcare providers and trusted and valued the recommendations they received [41, 59, 75, 76, 87]. Providers who supported women and fostered a safe clinical environment positively contributed to the use of screening services [41, 53, 56, 59, 65, 75, 76, 87]. Other key facilitators included the use of interpreters or interpretation services, [59, 65, 73, 75, 76] appointment reminders, [65, 75] and to have screening conducted by female care providers [56, 59, 65].

*Systemic*. Six studies indicated that a lack of health insurance and/or cost of services were key barriers to screening and vaccination, particularly in the USA and Australia [56, 59, 69, 70, 72, 87]. For example, 75% of Bhutanese-Nepali refugee women reported that their primary insurance was Medicaid/Medicare [53]. Rubens-Auguston et al., (2019) stated that HPV vaccinations should be publicly funded. Lack of or cumbersome transportation to services was reported as a barrier to testing sites [56, 59].

#### Access to sexual health education and information

*Individual*. Differences in sexual health education and literacy was the most commonly reported barrier influencing access to and use of SRH services [12, 13, 23, 41, 44, 45, 47, 49–51, 56, 57, 69, 70, 72–76, 92, 98, 102, 106]. For example, Ngum Chi Watts et al. (2015) reported that many young African-Australian mothers in their study sample were unaware that penile-vaginal intercourse could result in pregnancy—which contributed to unplanned pregnancies for some participants. Bolstering health literacy and empowering women with knowledge was seen as a key intervention [41, 49, 55, 59, 61, 63, 65, 70, 73, 78, 80, 87, 94, 98, 107]. Online [59, 80, 92, 94, 98], mobile [41], and social media platforms (e.g., YouTube, Facebook, Instagram; [41, 55, 59, 61, 94, 98] were suggested as modes of delivery for information, particularly around prevention, screening, and symptoms [41, 70]. Women in Hawkey at al. (2021), were particularly interested in less-often-discussed sexual health topics, and were interested in more information on sex, sexual desire and libido, vaginal pain, and consent and personal rights.

*Community*. Cultural and community stigma and shame surrounding SRH was one of the most prevalent barriers reported across studies, which limited knowledge sharing and access to education and services. Studies which sampled youth and/or parents reported a disconnect, with some parents feeling as though it was culturally inappropriate to educate their children on SRH, and youth felling like they couldn’t ask [12, 13, 45, 55, 74, 103]. Judgement and disapproval from family and peers contributes to feelings of fear and embarrassment around sexual health which contributes to lack of knowledge, unprotected sex, unplanned pregnancy, and the spread of STIs [13, 55, 71, 74]. While some parents still wanted to remove their children from sex education in schools, others were keen to reduce the shame and have open conversations with their children. Young women in four studies were keen to reduce the stigma around sexual health and exchange more intergenerational knowledge [13, 55, 74, 92]. A key barrier to this was that parents themselves may not have been educated in sexual health, so need support before they can educate their own children [92]. Gendered, culturally-appropriate workshops delivered in community on key SRH topics was recommended by 13 studies [41, 44, 55, 59, 61, 64, 65, 78, 92, 94, 98, 105, 107].

*Institutional*. Healthcare providers were viewed as valuable, trusted sources of information. Nine studies reported that women wanted SRH education from their clinicians [41, 59, 75, 76, 80, 87, 92, 98, 108]. However, two studies stated that appointment times are typically not long enough to accommodate the provision of clinical care and education, particularly if back and forth interpretation is also required [65, 87]. Additionally, a high proportion of studies reported that women did not receive any information on how to seek out and navigate SRH services in their host country and as such had difficulty locating and accessing care [47–49, 51, 56, 61, 64, 65, 70, 72, 73, 87, 94, 98]. Many studies noted that SRH education resources and materials must be made available in women’s preferred languages, which was often not the case [49, 55, 59, 61, 73, 78, 80, 87, 94, 98].

*Systemic*. 15 studies reported that the demands of the resettlement process often precede women’s own health concerns, especially SRH needs [44, 47, 50, 53, 56, 65, 69, 73–75, 78, 87, 92, 100, 102]. Women are often faced with more immediate demands, such as seeking affordable housing, enrolling their children in school, finding employment, and are more likely to be burdened with the additional needs of their children and family—often prioritizing their needs above their own. Additional structural barriers such as lack of childcare [47, 49, 61], limited access to transportation [49, 56, 57, 59, 61, 70, 86, 100], and their proximity to services [59, 70, 86, 100] hindered access and use.

## Discussion

To our knowledge, this is the first scoping review to comprehensively document the sexual and reproductive health inequities facing refugee and asylum-seeking women and the corresponding barriers to service use. Studies across the globe identified consistent evidence demonstrating inequitable access and use of sexual health services among refugee and asylum-seeking women across high-income countries. Refugee and asylum-seeking women are experiencing greater rates of unplanned pregnancy [89], significantly higher rates of abortion use [91, 95, 101] unmet contraceptive need [23, 85], low engagement with modern contraceptives [40, 86, 96, 99], and reduced rates of cervical cancer screening [49, 52, 53, 56, 58, 59]. These inequities are borne out of a myriad of interconnected individual, interpersonal/community, institutional, and structural barriers restricting access to services, supports, and education. This includes restricted pre-migration sexual health education, shame around women’s sexual functioning and family planning [44, 50, 92], sociocultural acceptance or normalization of GBV [44, 46, 63, 81], lack of interpretation services or interpreters in healthcare facilities [56, 75], socioeconomic constraints limiting access to care [70, 73], and systemic and interpersonal racism and xenophobia [53, 90, 100]. While refugees and asylum seekers are a diverse and heterogenous population with different countries of origin, languages, races, ethnicities, religions, and journeys through forced displacement, findings were similar enough across studies and populations to allow for theme development and comparison.

According to our results, individual and community/inter-personal factors were frequently discussed across studies, with an emphasis on the role stigma plays in sexual health. Stigma and shame surrounding sexual healthcare, particularly around birth control and abortion care, are not unique to refugee and asylum-seeking women; many individual and interpersonal factors discussed in this paper are not dissimilar to that affecting non-refugee women globally [109, 110]. This includes the global, gendered shame surrounding women’s sexual health and sexuality, which restricts women’s access to knowledge about their bodies and reproductive systems—all of which may have detrimental impacts on help-seeking, access to treatment, and effective family planning. Similarly, the persistence and sociocultural acceptance of gender-based violence remains a global concern, particularly for low-income, racialized, and/or queer women [111]. The dismantlement of systems of power that limit women’s autonomy and safety is critical and must be supplemented with personal and community-level education around sexuality, sexual health, and the rights of women [112, 113].

Reviewed studies revealed numerable institutional barriers to sexual health services for asylum seeking and refugee women. Results demonstrated a clear lack of accessible interpretation services in high-income countries, contributing to inaccessible care, failures to obtain informed consent, and poor procedural explanations [20, 90]. This subsequently resulted in maltreatment and mistrust for women accessing SRH care. Feelings of mistrust were reported throughout studies as a result of a lack of cultural competency and a history of forced or unsafe gynecological care [90]. Other studies recognized mistrust borne out of maltreatment and abuse women encountered by humanitarian aid workers, law enforcement and healthcare providers in transit [5, 6]. Exposure to trauma and sexual violence/exploitation in-transit necessitates culturally-safe and trauma-informed care to reduce further mistrust and violence [5, 6, 114]. While interpreters and interpretation services must be made available, culturally and linguistically congruent care providers are the gold-standard, removing the need for an intermediary entirely.

Results have demonstrated that despite living in wealthy, affluent nations with robust, comprehensive healthcare systems and services, refugee and asylum-seeking women face inequitable sexual healthcare. From a systems lens, anti-immigrant policies that restrict the livelihood and economic success of refugee and asylum-seeking families perpetuates poverty. For example, the failure to recognize internationally obtained education and employment credentials, costly re-certification programs, and low income-assistance rates force many families to live in poverty for an extended period. For example, in 2020, the poverty rate among refugees who had arrived after 2016 was nearly two-and-a-half times the rate of those born in Canada [115]. Income remains one of the most significant social dimensions of health, known to determine an individual or family’s health [116].

In countries without universal healthcare or public health insurance (e.g., USA), some families are left un- or under-insured, paying for many services out-of-pocket. Even in countries with universal care models (e.g., Canada, UK), non-citizens may receive differential health insurance with poorer coverage (see Canada’s Interim Federal Health Plan) [117, 118]. Those without extended health benefits may have to pay for additional services not covered by publilc insurance, such as prescription drugs (e.g., contraceptives, anti-viral or anti-biotic medication for STIs or STBBIs), physiotherapy for sexual dysfunction, or counselling and therapy for gender-based violence. Failure to provide comprehensive coverage for contraception is detrimental in nations where legal access to abortion is restricted or criminalized (e.g., USA, Poland) [119].

It is critical to recognize that most displaced persons have fled violence, instability, and persecution in the Global South (Venezuela, Syria, Sudan, Afghanistan)—thus most asylum-seekers and refugees are racialized [4, 120]. Most studies included in this review came from the USA, Australia, Canada, and Europe, all of which are majority-White states with long, problematic histories of colonization, slavery, and segregation, the legacies of which have created systemic inequities for racialized people which persist today [120–122]. Systems within these countries including criminal-legal systems, law enforcement, border services, healthcare, and social work, are intimately connected to, if not built on the exclusion of racialized people, and migrants [120, 123]. Systemic racism and xenophobia embedded within these systems perpetuates violence and trauma among racialized migrant communities, fostering many of the inequities reported in this review [124]. Healthcare providers, researchers, advocates, resettlement staff, and policy makers must work to recognize and advocate for the dismantling of systems of oppression.

### Strengths and limitations

This review identified studies examining access and use of sexual and reproductive health services among asylum-seeking and refugee women. The most significant challenge for this review was the inconsistent terminology used globally to describe refugee and asylum-seeking populations. For example, many studies lumped refugees under more general titles of ‘migrants’ or ‘immigrants;’ others labelled asylum-seekers as ‘undocumented people.’ When screening articles, it was difficult to discern whether some articles fit the inclusion criteria or not. As such, some articles may have been missed due to these differences or other inappropriately included in this review. Our team worked to thoroughly read through all articles included at the abstract and full-text screening to review the population understudy and determine if it fit with our inclusion criteria. Additionally, the quantitative and epidemiological research documenting differences in SRH service use between refugees/asylum-seekers and host-born women was lacking. The little evidence that was available was largely descriptive. As such, inferences made using quantitative research should be made with caution, given small sample sizes across included studies and differential uses of statistics across included studies.

### Directions for future research

The majority of research yielded by this systematic review was qualitative or mixed-methods with only descriptive statistics included for the quantitative portion. The limited quantitative data presented in this review should be interpreted with caution, given small sample sizes and differential statistics used across studies. To more concretely demonstrate the inequities described in this review, future epidemiological studies are needed to compare SRH outcomes between refugee women and women born in the resettlement country. This evidence may be essential when advocating for health system and policy change. Moreover, limited work was available on the experiences of asylum-seekers. Given their more precarious status, this population is at a greater risk for negative health outcomes. While this population has been historically difficult to reach, further research is essential to build health services and community programming that meets the unique needs of this highly marginalized group. Last, few, if any studies, examined the experiences of potentially more vulnerable women, such as those living with HIV/AIDS, LGBTQIA+ people, and sex workers. Refugee/asylum-seeking women with intersecting identities may face greater risks of experiencing negative SRH outcomes.

## Conclusions & implications for policy/practice

This systematic review has documented significant SRH inequities for refugee and asylum-seeking women. These results are paramount as the world continues to see unprecedented annual increases in the number forcibly displaced people worldwide. We posit a number of recommendations for policy, service delivery, intervention, and programming, based on facilitators acknowledged across included studies. At the individual and community level, we recommended education on SRH for refugees after arrival in their host country, in addition to know your rights workshops related to consent and gendered violence. This programming should be developed alongside essential community partners and/or leaders and be led by culturally and linguistically congruent healthcare or service providers. Interventions and programming embedded within and driven by community will be essential in addressing stigma and shame surrounding sexuality and SRH. At the institutional level, health systems must provide quickly accessible, free interpretation services where culturally and linguistically congruent care providers are not available. In-person interpreters were preferred, however, when this cannot be achieved, video interpretation services should be on stand-by. Policy-makers and educational institutions must employ specific policies (e.g., affirmative action) to ensure a diverse healthcare workforce that appropriately represents changing community populations. At the systems level, affordable childcare and comprehensive public transit will not only drastically improve women’s ability to attend essential health appointments, but more holistically support their social and economic independence. We also recommend the full coverage of contraceptives for all people, regardless of status, and the complete decriminalization of abortion.

## Supporting information

S1 Appendix Preferred Reporting Items for Systematic reviews and Meta-Analyses extension for Scoping Reviews (PRISMA-ScR) checklist. (DOCX)

S2 Appendix Search strategy [CINAHL, EBSCO]. (DOCX)
