# Supplementary material for: Access and use of sexual and reproductive health services among asylum-seeking and refugee women in high-income countries: A scoping review
Source: PLoS One. 2024 Nov 7;19(11):e0312746. doi: 10.1371/journal.pone.0312746 (PMC11542864; doi:10.1371/journal.pone.0312746)
Supplement: S2 Appendix — (DOCX) [file pone.0312746.s002.docx]

# Appendix II: Search strategy

**CINAHL (EBSCO)**

| **Search** | **Query** |
| --- | --- |
| 1 | woman OR women OR female OR mothers  *Unqualified search = searches title, abstract, and all subject words* |
| 2 | refugee* OR ( ((displaced OR resettled) W1 (person* OR people OR women OR woman)) ) OR newcomer* OR (asylum N1 seek*) |
| 3 | (MH "Contraception+") OR (MH "Contraceptive Agents+") OR (MH "Abortifacient Agents+") OR (MH "Papillomavirus Vaccine") OR (MH "AIDS Vaccines") OR (MH "Viral Hepatitis Vaccines+") OR (MH "Cervical Smears") OR (MH "Cervical Smears, Automated") OR (MH "Sexually Transmitted Diseases+") OR (MH "Family Planning+") OR (MH "Abortion, Induced+") OR (MH "Genital Diseases, Female+") OR (MH "Abortion, Criminal") OR (MH "Sexual Abuse+") OR (MH "Gender-Based Violence") OR (MH "Domestic Violence") OR (MH "Intimate Partner Violence") OR (MH "Sexuality+") OR (MH "Sexual Health")  *MH = CINAHL subject headings* |
| 4 | ((sexual OR women* OR reproducti*) N3 (health OR exam* OR abuse)) OR ((STI OR sexual* OR std OR pap OR cervical) N2 (screen* OR diagnos* OR test* OR smear*)) OR ( contracepti* OR "family planning" OR abortion ) OR ((hepatitis OR HPV OR papilloma) N2 (vaccin*) OR (sexual* N2 educat*) OR ((sexual* OR gender) W2 violence) OR rape* |
| 5 | S3 OR S4 |
| 6 | (MH "Women+") |
| 7 | S1 OR S6 |
| 8 | (MH "Refugees") |
| 9 | S2 OR S8 |
| 10 | Andorra OR Europe OR Austria OR Belgium OR Croatia OR Czech Republic OR Hungary OR Poland OR Slovakia OR Slovenia OR France OR Germany OR Gibraltar OR Greece OR Iceland OR Ireland OR Italy OR Liechtenstein OR Luxembourg OR Monaco OR Netherlands OR Portugal OR Spain OR United Kingdom OR Switzerland OR Canada OR United States OR Uruguay OR Chile OR Antigua OR Bahamas OR Barbados OR Puerto Rico OR Australia OR New Zealand OR Singapore OR Japan OR Hong Kong OR South Korea OR Denmark OR Scandinavia OR Bahrain OR Kuwait OR Oman OR Qatar OR United Arab Emirates OR Israel OR Bermuda OR Aruba OR Virgin Islands of the United States OR West Indies OR Brunei OR Cayman Islands OR Channel Islands OR Curacao OR Cyprus OR Estonia OR Faroe Islands OR Isle of Man OR Lithuania OR Macao OR Northern Mariana Islands OR San Marino OR Saudi Arabia OR Seychelles OR Sint Maarten OR St. Kitts and Nevis OR Saint Martin OR Taiwan OR Trinidad and Tobago OR Turks and Caicos Islands |
| 11 | \| (MH "Andorra") OR (MH "Europe") OR (MH "Austria") OR (MH "Belgium") OR (MH "Croatia") OR (MH "Czech Republic") OR (MH "Hungary") OR (MH "Poland") OR (MH "Slovakia") OR (MH "Slovenia") OR (MH "France") OR (MH "Germany+") OR (MH "Gibraltar") OR (MH "Greece") OR (MH "Iceland") OR (MH "Ireland") OR (MH "Italy") OR (MH "Liechtenstein") OR (MH "Luxembourg") OR (MH "Monaco") OR (MH "Netherlands") OR (MH Portugal") OR (MH "Spain") OR (MH "United Kingdom+") OR (MH "Switzerland") OR (MH "Canada+") OR (MH "United States+") OR (MH "Uruguay") OR (MH "Chile") OR (MH "Antigua") OR (MH "Bahamas") OR (MH "Barbados") OR (MH "Puerto Rico") OR (MH "Virgin Islands of the United States") OR (MH "Australia+") OR (MH "New Zealand") OR (MH "Singapore") OR (MH "Japan") OR (MH "Hong Kong") OR (MH "South Korea") OR (MH "Denmark") OR (MH "Scandinavia+") OR (MH "Bahrain") OR (MH "Kuwait") OR (MH "Oman") OR (MH "Qatar") OR (MH "United Arab Emirates") OR (MH "Israel") OR (MH "Bermuda") OR (MH "West Indies") OR (MH "Brunei") OR (MH "Mediterranean Islands") OR (MH "Estonia") OR (MH "Lithuania") OR (MH "Macao") OR (MH "San Marino") OR (MH "Saudi Arabia") OR (MH "Indian Ocean Islands") OR (MH "Taiwan") OR (MH "Trinidad and Tobago") \|  \| \| --- \| --- \| |
| 12 | S10 OR S11 |
| 13 | S5 AND S7 AND S9 AND S12 |
